# Molecular Docking Studies of a Cyclic Octapeptide-Cyclosaplin from Sandalwood

**DOI:** 10.3390/biom9110740

**Published:** 2019-11-15

**Authors:** Abheepsa Mishra, Satyahari Dey

**Affiliations:** 1 Plant Biotechnology Laboratory, Department of Biotechnology, Indian Institute of Technology Kharagpur, Kharagpur, West Bengal 721302, India; sdey12.iitkgp@gmail.com; 2 Department of Internal Medicine, The University of Texas Southwestern Medical Center, 5323 Harry Hines Blvd, Dallas, TX 75390, USA

**Keywords:** apoptosis, cyclosaplin, molecular docking, protein kinases, sandalwood

## Abstract

Natural products from plants, such as chemopreventive agents, attract huge attention because of their low toxicity and high specificity. The rational drug design in combination with structure-based modeling and rapid screening methods offer significant potential for identifying and developing lead anticancer molecules. Thus, the molecular docking method plays an important role in screening a large set of molecules based on their free binding energies and proposes structural hypotheses of how the molecules can inhibit the target. Several peptide-based therapeutics have been developed to combat several health disorders, including cancers, metabolic disorders, heart-related diseases, and infectious diseases. Despite the discovery of hundreds of such therapeutic peptides however, only few peptide-based drugs have made it to the market. Moreover, the in silico activities of cyclic peptides towards molecular targets, such as protein kinases, proteases, and apoptosis related proteins have not been extensively investigated. In this study, we explored the in silico kinase and protease inhibitor potentials of cyclosaplin, and studied the interactions of cyclosaplin with other apoptosis-related proteins. Previously, the structure of cyclosaplin was elucidated by molecular modeling associated with dynamics that were used in the current study as well. Docking studies showed strong affinity of cyclosaplin towards cancer-related proteins. The binding affinity closer to 10 kcal/mol indicated efficient binding. Cyclosaplin showed strong binding affinities towards protein kinases such as EGFR, VEGFR2, PKB, and p38, indicating its potential role in protein kinase inhibition. Moreover, it displayed strong binding affinity to apoptosis-related proteins and revealed the possible role of cyclosaplin in apoptotic cell death. The protein–ligand interactions using LigPlot displayed some similar interactions between cyclosaplin and peptide-based ligands, especially in case of protein kinases and a few apoptosis related proteins. Thus, the in silico analyses gave the insights of cyclosaplin being a potential apoptosis inducer and protein kinase inhibitor.

## 1. Introduction

Cancer is a well-recognized global health problem responsible for ∼7.6 million deaths (∼13% of all deaths) worldwide, which is expected to rise to 13.1 million by 2030 (WHO, 2012). Despite the advancements in the field of cancer research, there is still an urgency to discover and develop anti-cancer therapeutics. Natural products are of particular interest as chemopreventive agents because of their low toxicities and potential efficacies [1]. The conventional drug discovery techniques are time consuming and expensive processes [2]. Thus, rational drug design in combination with structure based modeling and rapid screening methods offer significant potential for identifying and developing lead anticancer molecules. The use of the molecular docking method addresses deducing the ligand binding sites with a protein of known three-dimensional structure. One of the computational approaches, docking, helps with screening a large set of molecules based on their free binding energies and proposes structural hypotheses of how the molecules could inhibit the target. For example, docking studies were used to select and rationally design novel biguanides towards m/hTAAR1 (Guariento et al., 2018) [3]. Additionally, docking studies were used to identify selective and 5-HT1A receptor agonists (Franchin et al., 2017) [4]. Recently, several in silico-based studies have been performed on small molecules, including peptides, to identify their anti-cancerous properties [5]. Peptide based therapeutics have been effective at combating several health disorders, including cancers, metabolic disorders, cardiovascular diseases, neurological disorders, kidney diseases, and infectious diseases. Peptides are structurally diverse, have a wide spectrum of therapeutic action, have low absorption in body tissues, and are highly specific to targets [6]. Several cyclic peptides with diverse biological activities, such as antibacterial activity, immunosuppressive activity, and anticancer activity, have been reported [7]. For example, tyrocidine and gramicidin S with antibacterial activity; cyclosporin A, displaying immunosuppressive activity; and Cyclo-RGDfV, with antiangiogenic activity [7,8,9]. Apart from their use as cytotoxic agents, peptides can also be used in drug formulations for enhancing biological activity, targeted drug delivery, or transport across cellular membranes. Thus, revival of interest in therapeutic peptides and extensive research has seen peptides entering into clinical trials improve significantly over the decade [10]. Despite the discovery of hundreds of such therapeutic peptides, however, only few peptide-based drugs have made it to the market. Moreover, thus far, the activities of cyclic peptides towards molecular targets such as protein kinases, proteases, and apoptosis related proteins, have never been explored. In this study we explorde the in silico kinase and protease inhibitor potentials of cyclosaplin and studied the interactions of cyclosaplin with other cancer-related proteins.

## 2. Materials and Methods

### 2.1. Softwares and Tools

ACD/ChemSketch 12.01, AutoDock Vina 1.1.2, Avogadro, CycloPsWeb, GROMACS, LigPlot, Modeller 9.2, MGL tools, Open Babel, Protein Data Bank (PDB), PubChem, PyMOL, and Swiss Target Prediction.

### 2.2. Ligand Preparation

The cyclic octapeptide (cyclosaplin) and various peptides (positive control) for specific proteins were used as ligands for docking studies (Table 1). The peptides (positive controls) were chosen based on their specific modes of action on cancer receptors. A molecular dynamics tool GROMACS and Modeller 9.2 program were used for the molecular modeling of cyclosaplin using appropriate energy minimization steps and simulations previously described [11,12]. Due to the unavailability of X-ray diffraction or NMR structure data for the chosen ligands, the ligand molecules were drawn in either ACD/Chem Basic freeware (ACD/ChemSketch 12.01) or using CycloPsWeb and saved as MDL mol file formats. The MDL files were converted to pdb format files using Open Babel. Further, all the ligand structures were energy-minimized using GROMACS and Modeller 9.2 program prior to docking studies [13]. The ligands used in the study are represented in the Table 1.

### 2.3. Lipinski Rule for Ligands

The peptide based-ligand molecules selected for docking experiments were screened for Lipinski’s rule of five. Lipinski’s rule of five [23] states that a drug molecule generally does not violate more than one of the following five rules

o Molecular mass less than 500 Da;
o High lipophilicity (expressed as LogP less than 5);
o Less than 5 hydrogen bond donors;
o Less than 10 hydrogen bond acceptors;
o Molar refractivity between 40 and 130.

Lipinski’s rule of five was also checked in Supercomputing Facility for Bioinformatics and Computational Biology, IIT Delhi, wherein PDB structures of the molecules were uploaded to the online server (http://www.scfbio-iitd.res.in/utility/LipinskiFilters.jsp).

### 2.4. Protein Preparation

Swiss Target Prediction was used to predicting the potential targets of cyclosaplin [24]. The protein structures were obtained from Protein Data Bank (PDB) [25]. The proteins selected for the study were epidermal growth factor’s receptor kinase domain (EGFR; PDB ID: 2GS2), vascular endothelial growth factor r2’s receptor kinase (VEGFR2; PDB ID: 1VR2), mitogen activated protein kinase (P38; PDB ID: 1P38), protein kinase B (PKB; PDB ID: 1GZN), phosphatase and tensin homolog tumor suppressor (PTEN; PDB ID:1D5R), matrix metalloproteases (MMP-2 (PDB ID: 1CK7), and MMP-9 (PDB ID: 1L6J)); and apoptosis related proteins (Procaspase 3 (PDB ID: 4JQZ), procaspase 7 (PDB ID: 1K88), caspase 9 (PDB ID: 2AR9), TRAIL (PDB ID: 1D2Q), and SURVIVIN (PDB ID: 1XOX)). EGFR kinase and procaspase 3 were previously used in our study and were used as controls in this study [11]. The files in pdb format for each receptor were converted to respective PDBQT format using MGL tools. The polar hydrogen atoms were added to the receptor molecules prior to docking studies. Three-dimensional affinity grids were created at the geometric centers of the target proteins.

### 2.5. Docking Studies Using AutoDock Vina

The energy-minimized structure of cyclic octapeptide and ligands o (positive control) were docked with target proteins using AutoDock Vina 1.1.2 [26]. The receptor and ligand files were represented in PDBQT file format, a modified pdb format containing atomic charges, atom type definitions for ligands, and topological information (rotatable bonds). For docking, the entire receptor was enclosed inside a grid box, with a grid spacing of 1 Å, keeping the receptor rigid and the ligand as a flexible molecule. The ligand’s backbone and side-chain were flexible and allowed to dock with the receptor to form all possible conformations. After defining the binding site and receptor–ligand preparation, docking runs were launched from the command prompt. The interaction energy between the ligand and the receptor was calculated for the entire binding site and expressed as affinity (kcal/mol).

### 2.6. Protein–Ligand Interactions

LigPlot was used to study protein–ligand interactions for a given pdb file encrypting the docking [27]. The LigPlot program self-generated schematic 2D representations of the interfaces of protein–ligand complexes from standard pdb file input. The output was in the form of informative representation of the intermolecular interactions and their strengths, including hydrogen bonds, hydrophobic contacts, and atom accessibilities. Hydrogen bonds are represented by dashed lines whereas hydrophobic contacts are depicted schematically. The amino acid residues of the protein involved with the above contacts are shown by an arc with spokes emerging towards the ligand atoms in contact and vice versa.

## 3. Results

### 3.1. Ligand Preparation

**Figure 1:**
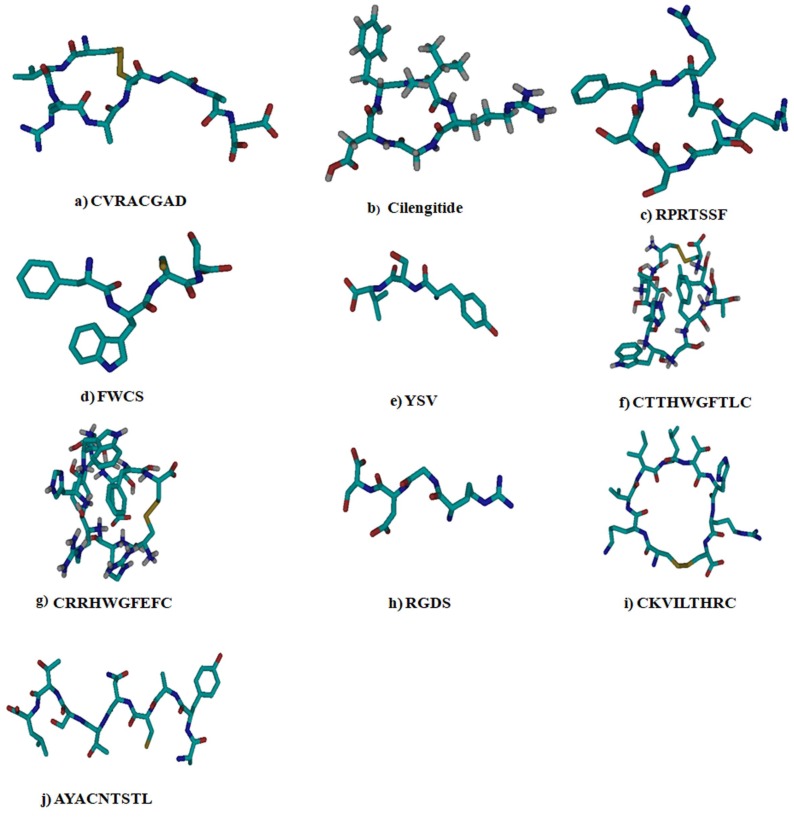
The different peptide-based ligands for targeting cancer-related proteins used in docking studies. Cyan blue = carbon, grey = hydrogen, deep blue = nitrogen, red = oxygen, and yellow = sulfur.

The ligand structures were drawn in CycloPsWeb or downloaded from PubChem and converted to pdb format using Open Babel and modeled using GROMACS and MODELLER 9.2 program along with cyclosaplin (Table 2). The structures were energy minimized and saved in PDBQT format by MGL tools (Figure 1). The energy minimized structure of cyclosaplin is represented in Figure S1.

### 3.2. Lipinski Rule

The ligands prepared for docking were screened for Lipinski’s rule of five. The commercially available or reported peptide inhibitors/inducers (positive control) were also tested against the target proteins respectively (Table 3).

### 3.3. Protein Preparation

**Figure 2:**
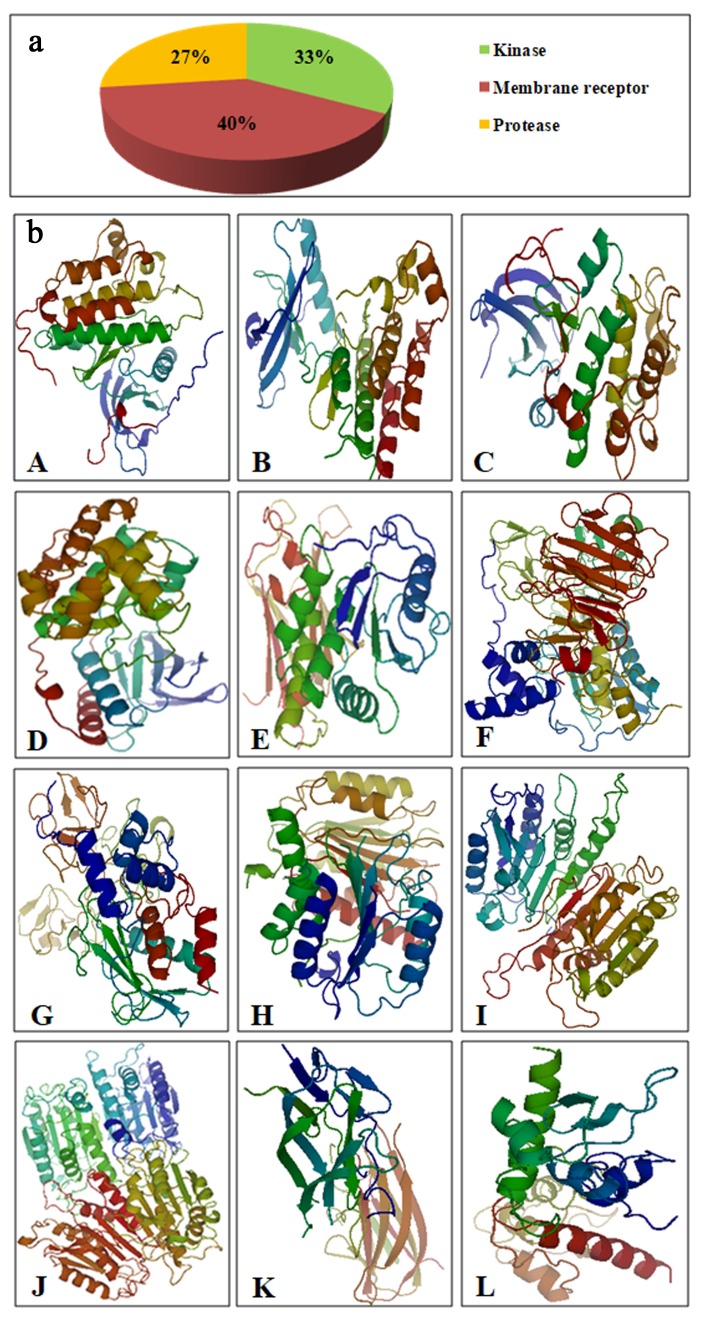
(**a**) Possible targets of cyclosaplin as predicted by Swiss Target Prediction. (**b**) Different energy minimized proteins (rainbow spectrum) used in docking studies. (**A**) EGFR kinase. (**B**) VEGFR2 kinase. (**C**) PKB. (**D**) p38. (**E**) PTEN. (**F**) MMP-2. (**G**) MMP-9. (**H**) Procaspase 3. (**I**) Procaspase 7. **(J**) Caspase 9. (**K**) TRAIL. (**L**) SURVIVIN.

Swiss Target Prediction was used to predict the possible targets of cyclosaplin (Figure 2a). A list of 50 potential targets was displayed, out of which top targets were selected from each class, such as kinases (33%), membrane receptors (40%), and proteases (27%). The cancer related proteins were downloaded from Protein Data Bank and (PDB) converted to PDBQT format using AutoDock tools (Figure 2b).

### 3.4. Docking Studies Using AutoDock Vina

**Figure 3:**
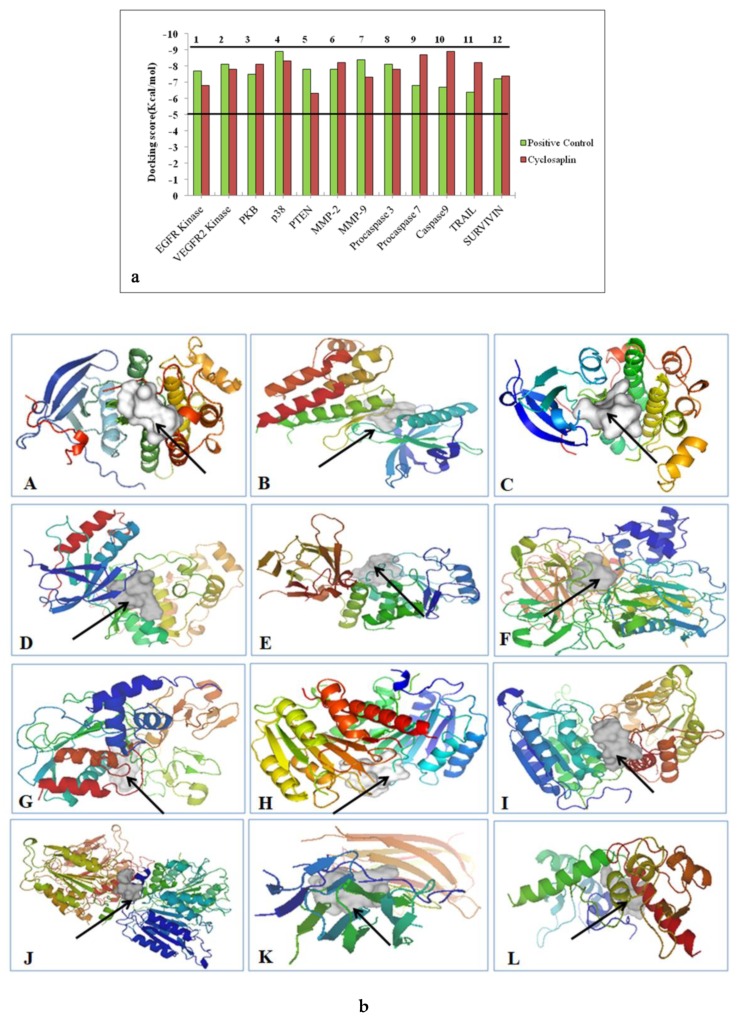
(**a**) Docking scores in kcal/mol for various cancer-related proteins. The binding affinities closer to 10 indicate efficient binding. (**b**) Cyclosaplin bound to different receptors. Cyclosaplin is shown in white, indicated by the arrows, and proteins are depicted with rainbow’s spectrum. (**A**) EGFR kinase. (**B**) VEGFR2 kinase. (**C**) PKB. (**D**) p38. (**E**) PTEN. (**F**) MMP-2. (**G**) MMP-9. (**H**) Procaspase 3 (previous study [11]). (**I**) Procaspase 7. (**J**) Caspase 9. (**K**) TRAIL. (**L**) SURVIVIN.

**Figure 4:**
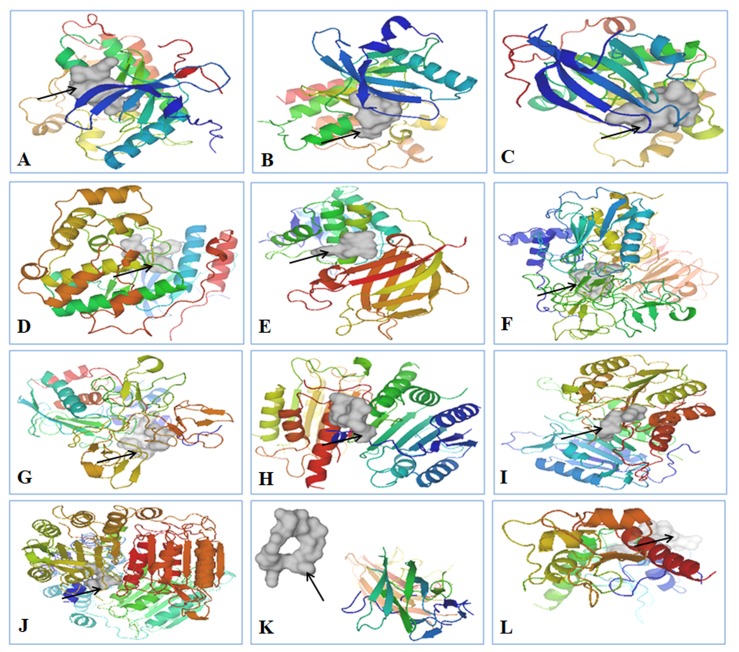
Peptide based ligands bound to specific proteins. Ligands are shown in white, indicated by the arrows, and proteins are depicted in rainbow spectrum. (**A**) CVRACGAD bound to EGFR kinase, (**B**) Cilengitide bound to VEGFR2 kinase, (**C**) RPRTSSF bound to PKB, (**D**) FWCS bound to p38, (**E**) YSV bound to PTEN, (**F**) CTTHWGFTLC bound to MMP-2, (**G**) CRRHWGFEFC bound to MMP-9, (**H**) Cilengitide bound to Procaspase 3, (**I**) RGDS bound to Procaspase 7, (**J**) RGDS bound to Caspase 9, (**K**) CKVILTHRC unbound to TRAIL, and (**L**) AYACNTSTL bound to SURVIVIN.

The docking process was carried out using AutoDock Vina. The docking scores are graphically represented in Figure 3a and the binding affinities of ligands are represented as kcal/mol (Table 4). The affinity value of less than or closer to 5 kcal/mol depicts negligible binding, whereas values closer to 10 kcal/mol indicate efficient binding (Figure 3a). The protein–cyclosaplin docking studies are represented in Figure 3b, and protein–ligand interactions (positive control) are represented in Figure 4. Cyclosaplin exhibited stronger binding affinity (>5 kcal/mol for all the cancer-related proteins).

### 3.5. Protein–Ligand Interactions

**Figure 5:**
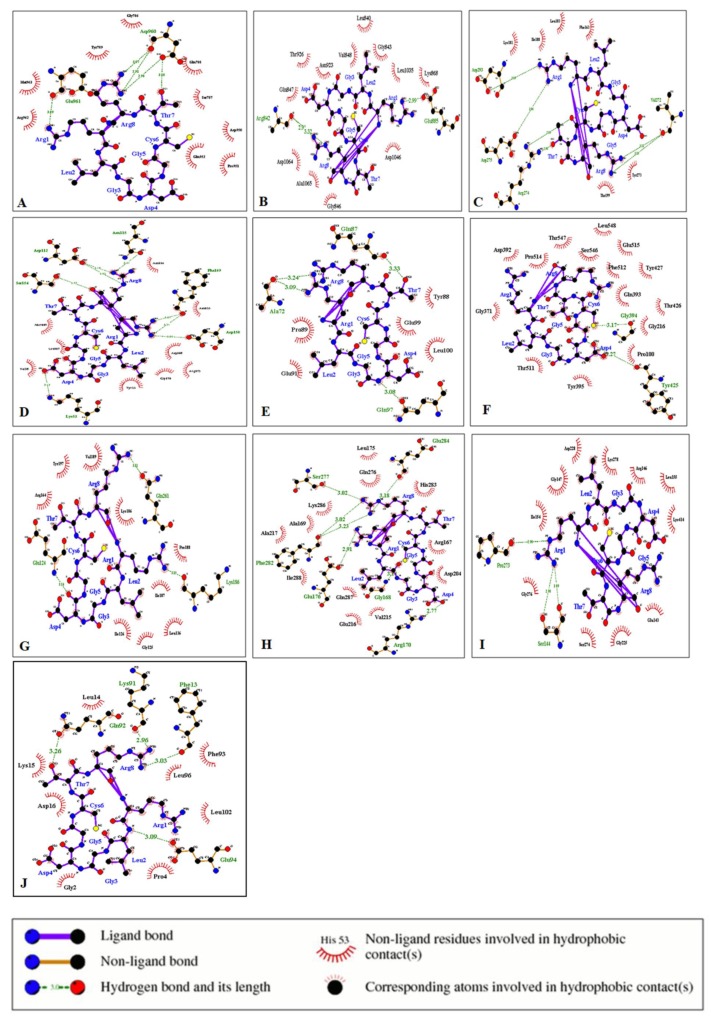
Interaction of cyclosaplin with various cancer-related proteins. (**A**) EGFR Kinase. (**B**) VEGFR2 Kinase. (**C**) PKB. (**D**) p38. (**E**) PTEN. (**F**) MMP-2. (**G**) Procaspase 3. (**H**) Procaspase 7. (**I**) Caspase 9. (**J**) SURVIVIN.

**Figure 6:**
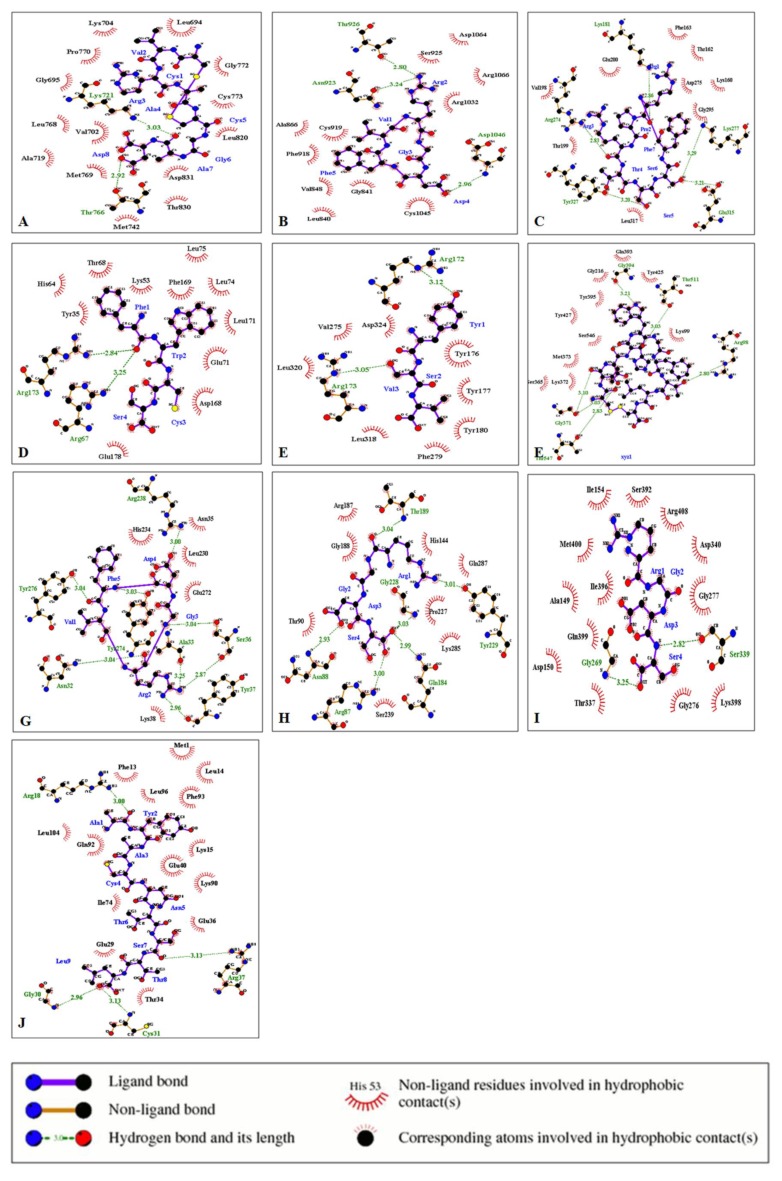
Protein–ligand interactions using LigPlot. (**A**) CVRACGAD and EGFR kinase. (**B**) Cilengitide and VEGFR2 kinase. (**C**) RPRTSSF and PKB. (**D**) FWCS and p38. (**E**) YSV and PTEN. (**F**) CTTHWGFTLC and MMP-2. (**G**) Cilengitide and Procaspase 3. (**H**) RGDS and Procaspase 7. (**I**) RGDS and Caspase 9. (**J**) AYACNTSTL and SURVIVIN.

The protein–ligand interaction study was performed using LigPlot. The interactions of the ligands cyclosaplin and various peptide-based ligands with amino acids residues of the target proteins are shown in Table 5. The H-bonds and hydrophobic contacts between the docked complexes are shown in Figure 5 and Figure 6.

## 4. Discussion

Peptides are effective receptor-binding ligands; many other classes of ligands sharing this binding trait include small molecules, endogenous proteins, and antibodies [28]. Cyclic peptides have built-in, stable pharmacokinetic characteristics, including enzyme stability, conformational rigidity, improved receptor site selectivity, and pharmacological specificity. In addition, cyclic peptides are reported to be potent protein kinase inhibitors, protease inhibitors (MMP-2 and MMP-9), angiogenesis blockers, and apoptosis inducers [29,30,31,32]. In comparison to small molecules, cyclic peptides can be more selective, whereas the size of molecules can be less than protein molecules such as antibodies and growth factors. Therefore, in the present study, an attempt was made to investigate the potential of cyclosaplin and other reported peptide-based ligands (positive control) against specific cancer-related proteins.

In our previous study, cyclosaplin was isolated, purified, and characterized from *Santalum album* L. [11]. The cyclosaplin was molecularly modeled and the energy minimized structure was further used for docking studies (Figure S1). The ligands were energy minimized prior to docking studies (Table 1 and Table 2, Figure 1). All of the peptide-based ligands, along with cyclosaplin, were screened for Lipinski’s rule of five (Table 3). Some of these peptides violated the rules, yet displayed drug-like properties in the experimental studies in vitro. Cyclic peptides tend to have properties (e.g., MW, number of polar atoms, and total polar surface area) that put them outside conventional predictors of “drug-likeness,” such as Lipinski’s rule of five [23]. In spite of this, many compounds exhibited drug-like properties, including the potential to penetrate cellular membranes.

The potential targets of cyclosaplin were predicted by Swiss Target Prediction [23] (Figure 2a) and the proteins used in docking studies were energy minimized, which is represented in Figure 2b. Relative binding affinities were scored for the cyclosaplin and peptide-based ligands, represented as kcal/mol (Table 4). The affinity value of less than five depicts negligible binding, whereas values closer to 10 kcal/mol indicate efficient binding. In addition, the docking scores for various cancer-related proteins was represented graphically, as shown in Figure 3. Docking studies revealed the strong binding affinities of cyclosaplin towards apoptosis-related proteins procaspase 3 (−7.8 kcal/mol; [11]), procaspase 7 (−8.7 kcal/mol), caspase 9 (−8.9 kcal/mol), TRAIL (−8.2 kcal/mol), SURVIVIN (−7.4 kcal/mol), and protease MMP-2 (−8.2 kcal/mol) (Figure 3a,b). Cyclosaplin also demonstrated effective binding affinities towards other cancer-related proteins, such as EGFR (−6.8 kcal/mol) [9], VEGFR2 (−7.8 kcal/mol), PKB (−8.1 kcal/mol), p38 (−8.3 kcal/mol), PTEN-tumor suppressor (−6.3 kcal/mol), and MMP-9 (−7.3 kcal/mol) (Table 4, Figure 3). The peptide-based ligands (positive control) reported in the literature or under clinical studies showed strong binding affinities with the specific proteins except for TRAIL (Figure 3). In case of TRAIL, the ligand remained unbound to the protein with a score of −6.4 kcal/mol. The result indicated the possible role of cyclosaplin in mediating apoptotic cell death. Cyclosaplin exhibited stronger binding affinity (>5 kcal/mol for all the protein targets which is consistent with our previously shown experimental study were we have shown that the cyclosaplin exhibits significant anti-proliferative activity with an IC_50_ 2.06 µg/mL in MDA-MB-231 cells (Mishra et al., 2014).

In contrast to most small molecule drugs, peptides have high affinity, strong specificity for targets, and low toxicity, whereas, in contrast to chemotherapeutics antibodies, they have good penetration of tissues because of their small size [33,34,35,36]. Cyclization is also thought to minimize conformational entropy losses upon target binding, although some studies have shown the impact of cyclization on binding entropy to be more complex [37].

The interaction of the cyclosaplin and other peptide-based ligands (positive control) with the amino acids of various cancer-related proteins were also determined (Table 5). We previously showed the structure–activity relationship for EGFR kinase with cyclosaplin [11], but in the present study, we demonstrated the possible interactions between protein and ligand with key amino acid residues involved in such interactions. In case of EGFR kinase, the peptide inhibitor CVRACGAD (cyclic) showed no similar interactions with cyclosaplin for amino acid residues of the protein (Table 5). The cyclosaplin interacted with Asp 960/Glu961 and Ser787/Tyr789, forming H-bonds and hydrophobic contacts respectively (Table 5, Figure 5A and Figure S2). Asp-960/Glu-961 facilitates the movement of the C-terminal tail of the EGF receptor to regulate asymmetrical dimer formation [27]. The side chain of Asp-960 interacts with that of Ser787, and mutation at this site enhanced protein kinase activity [38], whereas Tyr789 is the site for phosphorylation, the new potential binding site from the catalytic domain of EGFR [39]. The positive control CVRACGAD (Figure 6A and Figure S2B) forms a H-bond with Lys 721, whose side chains interact with the ATP forming salt-bridges in activated kinases [40]. In addition, it interacts with the glycine-rich nucleotide phosphate-binding loop (Gly695-Gly700) and DFG motif (Asp831-Gly833) within the A-loop [40]. The interaction between cyclosaplin and EGFR kinase occurs on Asp960, Glu961, Ser787, and Tyr789 with significant binding affinity. The residues mentioned above played a key role in dimer formation and the site for phosphorylation, highlighting that cyclosaplin could inhibit EGFR kinase by interacting with C-terminal region of EGFR (Table 5, Figure 5A). Previously, we have shown binding of cyclosaplin to EGFR through co-localization studies resulting in the sensitization of MDA-MB-231 cells and the induction of apoptosis [11]. Interestingly, certain common amino acid residues of most of the proteins shared trans-similarity; for example, residues involved in H-bond formation in cyclosaplin matched with residues forming hydrophobic contacts in peptide-based ligands. It is not necessary for hydrophobic interactions to occur only between the amino acids with hydrophobic side chains. It can occur between all the amino acid residues depending on their total degrees of hydrophobicity [41]. The architecture of VEGFR-2 involves several important loop domains, including glycine-rich loop (also refers to nucleotide binding loop) at residues 841–846, the catalytic loop at residues 1026–1033, and the activation loop at residues 1046–1075 [42]. The active sites around the ATP-binding domain of VEGFR-2 consist of three hydrophobic regions (regions 1–3) and one polar region (region 4). Between region 1 and the region 2, Lys866, Glu883, and Asp1044 are crucial for receptor activation [42]. Region 3 contains only a few residues, including Leu838 and Phe916. The unique polar region involves several residues, such as Asn921, Cys1043, Arg1030, and Asn1031 [43]. The interaction between cyclosaplin and VEGFR2 occurs on Glu 885, Asn923, Asp1046, Cys919, and Lys868 with strong binding affinity indicating that cyclosaplin could inhibit VEGFR-2 activity by interacting with the ATP-binding site of VEGFR-2 (Table 5, Figure 5B, Figure 6B, and Figure S2). A similar residual interaction occurred in the case of antiangiogenic peptide, cilengitide, and VEGFR2 kinase. We envisage that a fixed geometry ascertained due to cyclization in peptides could help it bind to receptors more effectively. The RGD peptide or RGD-like peptides are good examples of cyclic peptides as receptor binding molecules (Figure S4). The binding affinity of cyclosaplin towards α5β3 was closer to 10 kcal/mol (−9.5 kcal/mol), indicating strong binding (Table S1). Some common amino acid residues, such as Arg274, Asp275, Lys181, Phe163, and Thr199 of PKB interacted with both cyclosaplin (RLGDGCTR) and RPRTSSF (Table 5, Figure 5C, Figure 6C, Figures S2 and S3). Mutational analysis of Arg274 in Akt2 is essential for shielding Ther308 in the activation loop against dephosphorylation [44]. The α-helix at C-terminal (αC helix) of the N lobe plays a vital role in regulating the catalytic functions in all the protein kinases [45]. In the inactive state of PKB, His 196, and Glu 200 of the αC helix are disordered, and contacts between Glu 200 and Lys 181, and those between His 196 and pThr 309, are not formed [45]. The interaction between cyclosaplin and PKB occurs on Arg274, Lys181, Phe163, Thr199, Tyr273, and Leu183 with strong binding, indicating its possible role as PKB inhibitor (Table 5, Figure 5C). Moreover, the above interacting amino acid residues are also common to RPRTSSF, the positive control used in this study (Figure 6C). In p38, both the peptide-based ligands (FWCS and cyclosaplin) had interactions with common amino acid residues involved in phosphate and ATP binding sites (Table 5, Figure 5D, Figure 6D, Figures S2 and S3). Among all of the MAP kinases, the phosphorylation sites (Thr-180 and Tyr-182), and the putative phosphate binding ligands (Arg67, Arg70, Arg149, Arg173, Arg186, and Arg189) are conserved in homologous positions, and thus, may interact similarly in different active MAP kinases [46]. The available structural data revealed that most of the small molecule inhibitors of protein kinases bind in the ATP binding pocket [46,47]. ATP binding sites of p38 are the residues corresponding to Glu71, Lys53, and Asp168 [48]. Several of these kinases play a crucial role in cellular proliferation and migration. A scratch assay or cell migration assay was performed in MDA-MB-231 cells with and without cyclosaplin treatment (10 µg/mL) for 0 and 12 h (Figure S5). Cyclosaplin abrogated cellular migration of MDA-MB-231 cells effectively, suggesting the kinases being down-regulated.

Some of the amino acids of MMP-2 interacted with both the peptide ligands (CTTHWGFTLC and cyclosaplin), forming H-bonds and hydrophobic contacts (Figure 5F and Figure 6F). Similarly, in the case of procaspase 7 (Figure 5H and Figure 6H), caspase 9 and survivin (Figure 5 and Figure 6) a few amino acids shared similar interactions with both the peptide-based ligands (positive control; cyclosaplin).

In caspase 9 (Figure 5I and Figure 6I), Gly276 interacted with both RGDS and cyclosaplin, forming hydrophobic contacts, whereas in surviving, Gln92, Phe13, Phe93, Leu14, Leu96, and Lys15 formed interactions with both the peptide based ligands (Figure 5J and Figure 6J). No common interactions were observed in cases of PTEN, MMP-9, and Procaspase 3 (Figure 5 and Figure 6), whereas the positive control failed to interact with TRAIL. Additionally, the apoptotic mechanism was further validated by analyzing the alterations in the mRNA expression of Bax and caspase 3, using GAPDH as control. An upregulation in the mRNA expression of caspase 3 occurred after cyclosaplin treatment in MDA-MB-231 cells (Figure S6a). Similarly, the level of Bax, proapoptotic gene increased in cyclosaplin induced MDA-MB-231 cells after 24 h of treatment (Figure S6a). There was an approximately two-fold increase in mRNA levels of caspase 3 and Bax (Figure S6b). In addition to this, we showed in an earlier study, the induction of apoptosis in MDA-MB-231 cells through caspase-3 activity [11]. Arg1 and Arg8 of cyclosaplin interacted well with the amino acid residues of cancer-related proteins. This could have been possible because Arg side chains provide positive charges as well as hydrogen bonding capabilities to attract the peptide to the negative surface charges of the protein. The binding affinity of cyclic peptide TYY, along with its interaction with EphA4 receptor tyrosine kinase by using AutoDock 4 and LigPlot have been reported elsewhere [49].

Previously, in a 3D cell culture, the efficacy of cyclosaplin was shown as an anticancer agent [50]. Apart from anticancer activity, the other biological activities, such as antimicrobial activity, antiviral activity, and immunomodulatory function, need to be investigated for cyclosaplin. In this context, several analogs of cyclosaplin should be designed and screened in silico for the above-mentioned biological activities prior to in vitro studies. Thus, the in silico experiments gave a clear insight of cyclosaplin potential as an apoptosis inducer and a potential protein kinase inhibitor.

## 5. Conclusions

The structure of the cyclic octapeptide was elucidated previously by molecular modeling associated with dynamics and was used in the docking studies. Docking studies showed the strong affinity of cyclosaplin towards cancer-related proteins, especially protein kinases and apoptosis-related proteins. Thus, the in silico analyses revealed the potential of cyclosaplin as an apoptosis inducer and a protein kinase inhibitor. Based on these studies, appropriate in vitro and in vivo experiments should be designed rationally to validate its biological activity.

## Figures and Tables

**Table 1:** Ligands used in the study.

S.No.	Ligand	References
1	CVRACGAD (Cyclic)	[14]
2	Cilengitide (Cyclic)	[15]
3	RPRTSSF (Cyclic)	[16]
4	FWCS (Linear)	[17]
5	YSV (Linear)	[18]
6	CTTHWGFTLC (Cyclic)	[19]
7	CRRHWGFEFC (Cyclic)	[19]
8	RGDS (Linear)	[20]
9	CKVILTHRC (Cyclic)	[21]
10	AYACNTSTL (Linear)	[22]
11	Cyclosaplin (Cyclic)	[11]

**Table 2:** Molecular weight and molecular formulae of the ligands.

S.No.	Ligand	Molecular Weight (Da)	Molecular Formula
1	CVRACGAD (Cyclic)	791.9	C_29_H_49_N_11_O_11_S_2_
2	Cilengitide (Cyclic)	588.6	C_27_H_40_N_8_O_7_
3	RPRTSSF (Cyclic)	875.0	C_39_H_66_N_14_O_9_
4	FWCS (Linear)	541.6	C_26_H_31_N_5_O_6_S_1_
5	YSV (Linear)	367.4	C_17_H_25_N_3_O_6_
6	CTTHWGFTLC (Cyclic)	1166.3	C_52_H_71_N_13_O_14_S_2_
7	CRRHWGFEFC (Cyclic)	1338.5	C_60_H_79_N_19_O_13_S_2_
8	RGDS (Linear)	433.4	C_15_H_27_N_7_O_8_
9	CKVILTHRC (Cyclic)	1070.3	C_45_H_79_N_15_O_11_S_2_
10	AYACNTSTL (Linear)	943.0	C_39_H_62_N_10_O_15_S_1_
11	Cyclosaplin (Cyclic)	858.9	C_33_H_60_N_14_O_12_S_1_

**Table 3:** Physiochemical parameters of ligand molecules screened for Lipinski’s rule.

Ligand	Molecular Weight (Da)	Hydrogen Bond Donor	Hydrogen Bond Acceptor	LogP	Molar Refractivity	Rules Satisfied
CVRACGAD	791.9	13	13	−4.7	206.6	1/5
Cilengitide	588.6	7	8	−1.4	170.9	2/5
RPRTSSF	875.0	15	12	−5.9	236.7	0/5
FWCS	541.6	8	7	−0.7	143.2	2/5
YSV	367.4	6	6	−1.0	93.14	4/5
CTTHWGFTLC	1166.3	16	17	−4.0	331.1	1/5
CRRHWGFEFC	1338.5	20	17	−3.6	381.6	1/5
RGDS	433.4	10	8	−4.7	99.7	4/5
CKVILTHRC	1070.3	16	16	−3.3	306.4	1/5
AYACNTSTL	943.0	16	16	−6.1	230.0	0/5
Cyclosaplin	858.9	17	13	−6.5	243.0	0/5

**Table 4:** Comparative binding affinity of different ligands with receptors.

S.No.	Receptor	Ligand	Binding Affinity (kcal/mol)
1	Epidermal Growth Factor Receptor Kinase	CVRACGAD Cyclosaplin	−7.7 −6.8
2	Vascular Endothelial Growth Factor r 2 Receptor Kinase	Cilengitide Cyclosaplin	−8.1 −7.8
3	Protein Kinase B	RPRTSSF Cyclosaplin	−7.5 −8.1
4	p38 (Mitogen Activated Protein Kinase)	FWCS Cyclosaplin	−8.9 −8.3
5	PTEN	YSV Cyclosaplin	−7.8 6.3
6	Matrix metalloproteinase-2 (MMP-2)	CTTHWGFTLC Cyclosaplin	−7.8 −8.2
7	Matrix metalloproteinase-9 (MMP-9)	CRRHWGFEFC Cyclosaplin	−8.4 −7.3
8	Procaspase 3	Cilengitide Cyclosaplin	−8.1 −7.8
9	Procaspase 7	RGDS Cyclosaplin	−6.8 −8.7
10	Caspase 9	RGDS Cyclosaplin	−6.7 −8.9
11	TRAIL	CKVILTHRC Cyclosaplin	−6.4 −8.2
12	SURVIVIN	AYACNTSTL Cyclosaplin	−7.2 −7.4

**Table 5:** Molecular interactions of ligands with amino acids of proteins (amino acids showing similar interactions are marked in bold; black = cis similarity; red = trans similarity).

S.No.	Protein	Ligand	Hydrophilic Interactions	Hydrophobic Contacts	No. of H-Bonds
1	EGFR Kinase	CVRACGAD	Lys721, Thr766	Ala719, Asp831, Gly695, Gly772, Leu694, Leu768	2
		Cyclosaplin	Glu961, Asp960 (4)	Arg962, Asp950, Gln788, Gln952, Gly786, Met963, Pro951, Ser787, Tyr789	5
2	VEGFR 2 Kinase	Cilengitide	**Asn923**, **Asp 1046**, **Thr926**	Ala866, Arg1032, Arg1066, **Asp1064**, Cys919, Cys1045, Gly841, **Leu840**, Phe918, Ser925, **Val848**	3
		Cyclosaplin	Arg842, Arg842, Glu885	Ala1065, **Asn923**, **Asp1046**, **Asp1064**, Gly843, Gly846, Gln847, **Leu840**, Leu1035, Lys868, **Thr926**, **Val848**	3
3	Protein Kinase B	RPRTSSF	**Arg274**, Glu315, **Lys181**, Lys277, Tyr327	**Asp275**, Glu200, Gly295, Leu317, Lys160, **Phe163**, Thr162, **Thr199**, Val198	5
		Cyclosaplin	**Arg274**, Arg274, **Asp275**, Asp293, Val272, Val272	Ile188, Leu183, **Lys181**, **Phe163**, **Thr199**, **Tyr273**	6
4	p38 (Mitogen Activated Protein Kinase)	FWCS	**Arg173**, Arg 67	**Asp168**, Glu71, Glu178, His64, Leu74, Leu75, Leu171, **Ly53**, **Phe169**, Thr68, **Tyr35**	2
		Cyclosaplin	Asp112, Asp112, Asp150, Asn115, **Lys53**, **Phe169**, **Phe169**, Ser154	**Arg173**, Asn114, Asn155, **Asp168**, **Gly170**, Leu167, Met109, **Tyr35**, Val38	8
5	PTEN	YSV	Arg172, Arg173	Asp324, Leu318, Leu320, Phe279, Tyr176, Tyr177, Tyr180, Val275	2
		Cyclosaplin	Ala72, Ala72, Gln87, Gln97	Glu91, Glu99, Leu100, Pro89, Tyr88	4
6	MMP-2	CTTHWGFTLC	Arg98, **Gly371**, **Gly394**, **Thr 511**, Thr547	**Gln393**, **Gly216**, Lys99, Lys372, Met373, Ser365, **Ser546**, Tyr395, **Tyr425**, **Tyr427**	6
		Cyclosaplin	**Gly394**, **Tyr425**	Asp392, Glu515, **Gln393**, **Gly216**, **Gly371**, Phe512, Pro100, Pro514, Leu 548, **Ser546**, Thr426, **Tyr427**, **Thr511**, Tyr277, Tyr395	2
7	MMP-9	CRRHWGFEFC	Leu371, Arg2, Cys1	Arg370, Arg424, Glu427, Gln391, Gly392, Lys92, Phe425, Pro97, Pro233, Ser240, Ser242, Thr426 Tyr393, Tyr423	3
		Cyclosaplin	Arg221, Thr331	Arg279, Asp226, Asp284, Gly227, Gly285, Pro219, Pro272, Thr220,	4
8	Procaspase 3	Cilengitide	Ala33, Arg238, Asn32, Ser36 (2), Tyr37, Tyr274, Tyr276	Asn35, Glu272, Leu230, Lys38, His234	8
		Cyclosaplin	Gln261, Glu124, Lys186	Arg164, Gly125, Ile126, Ile187, Leu136, Lys186, Pro188, Tyr197, Val189	3
9	Procaspase 7	RGDS	Arg87, Asn88, Gly228, Gln184 Thr189, Tyr229	Arg187, Gly188, **Gln287**, His144, Lys285, Pro227, Ser239, Thr90	6
		Cyclosaplin	Arg170, Glu176, Glu284, Gly168, Phe282, Phe282, Ser277	Ala169, Ala217, Arg167, Asp204, Gln276, Lys286, Leu175, **Gln287**, Ile288, Val215, Glu216, His283	7
10	Caspase 9	RGDS	Gly269, Ser339	Ala149, Arg408, Asp150, Asp340, **Gly276**, Gly277, Gln399, Ile154, Ile396, Lys398, Met400, Thr337	2
		Cyclosaplin	Pro273, Ser144, Ser144	Arg146, Asp228, Glu143, Gly147, Gly225, **Gly276**, Ile154, Leu155, Lys278, Lys414, Ser274	3
11	SURVIVIN	AYACNTSTL	Arg18, Arg37, Cys31, Gly30	Glu29, Glu36, Glu40, **Gln92**, Ile74, **Leu14**, **Leu96**, Leu104, Lys15, Lys90, Met1, **Phe13**, **Phe93**, Thr34	4
		Cyclosaplin	**Gln92**, Glu94, Lys91, **Phe13**	Asp16, Gly2, **Leu14**, Leu96, Leu102, **Lys15**, **Phe 93**, Pro4	4

## Supplementary Materials

The supplementary materials are available online at https://www.mdpi.com/2218-273X/9/11/740/s1.
