# Supplementary material for: Molecular Docking Studies of a Cyclic Octapeptide-Cyclosaplin from Sandalwood
Source: Biomolecules. 2019 Nov 15;9(11):740. doi: 10.3390/biom9110740 (PMC6920920; doi:10.3390/biom9110740)
Supplement: Supplementary file 1 [file biomolecules-09-00740-s001.pdf]

Supplementary Figures

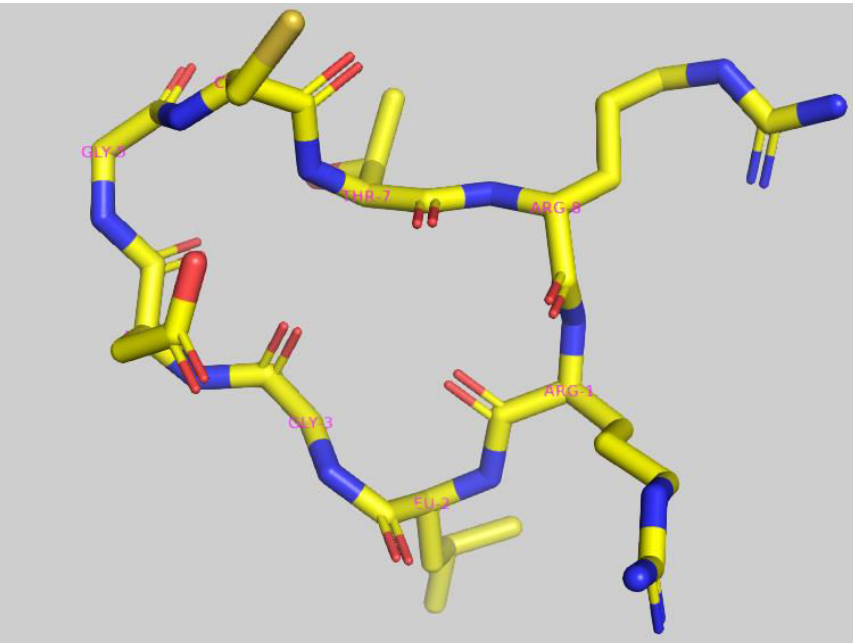

Fig. S1 The energy minimized structure of cyclosaplin

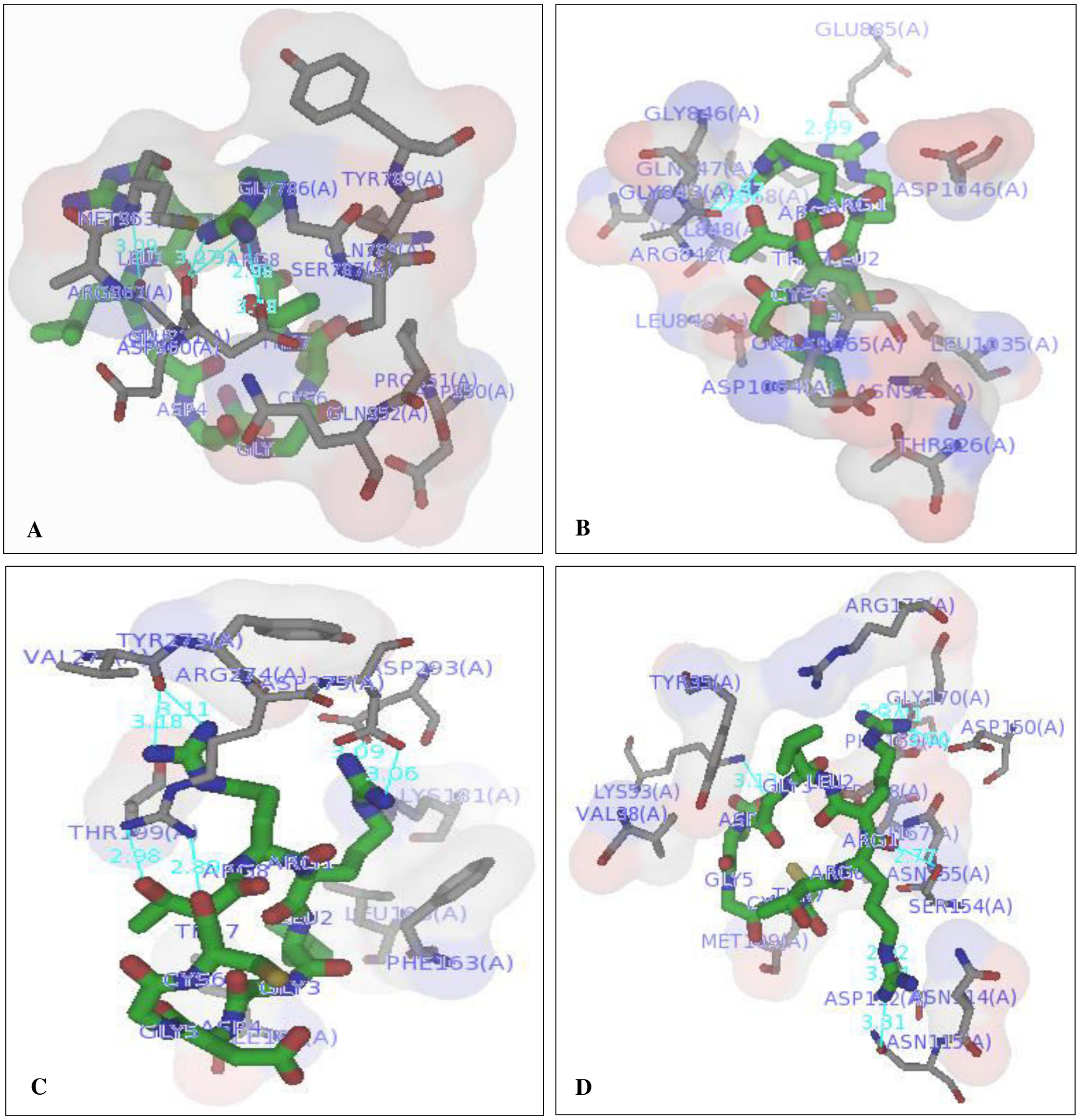

Fig. S2 Interaction of cyclosaplin with various cancer-related proteins using LigPlot (3D representation). A) EGFR Kinase B) VEGFR2 Kinase C) PKB D) p38.

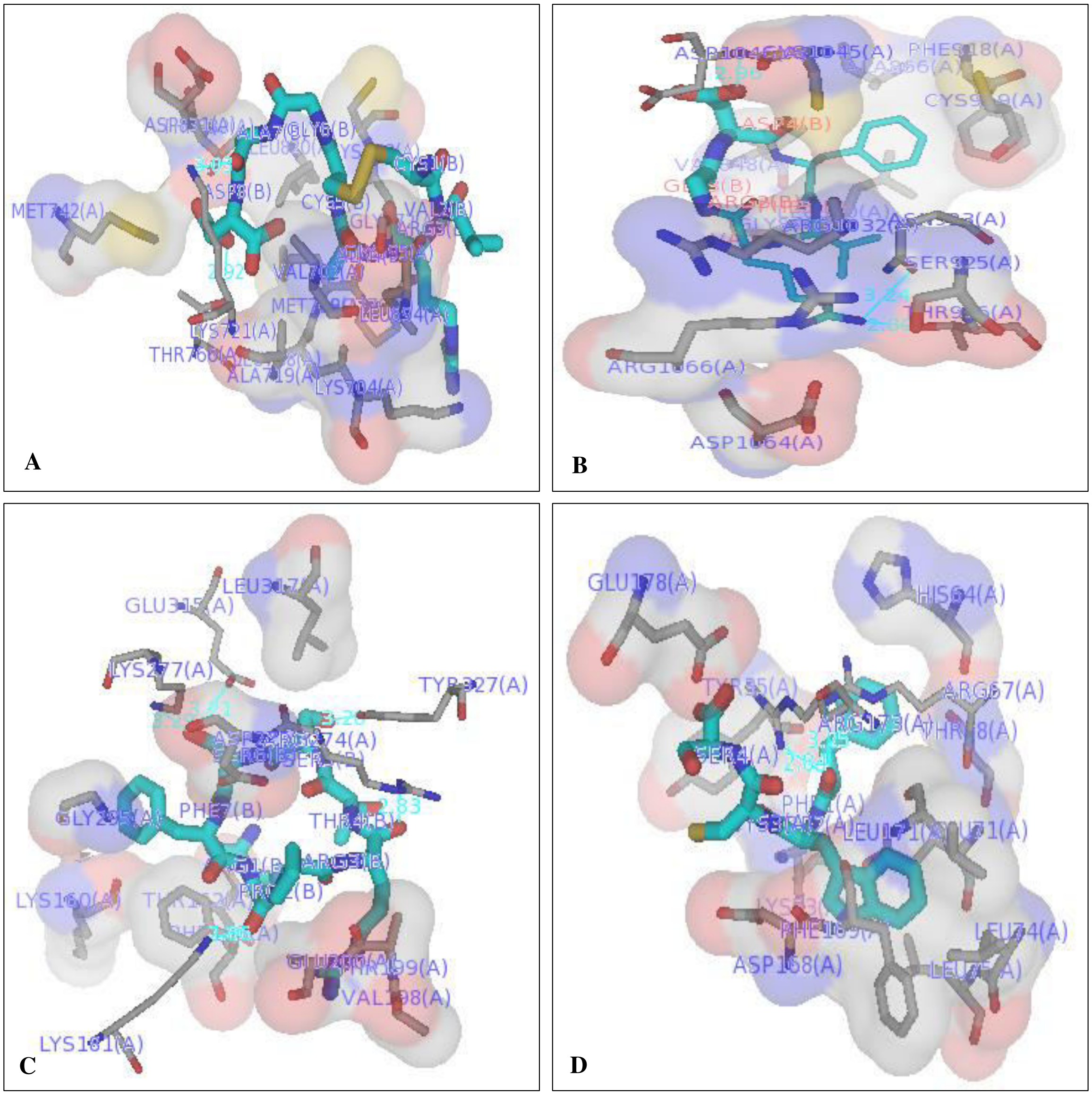

Fig. S3 Interaction of a few peptide-based ligands with various cancer-related proteins using LigPlot (3D representation). A) CVRACGAD and EGFR kinase B) Cilengitide and VEGFR2 kinase C) RPRTSSF and PKB D) FWCS and p38.

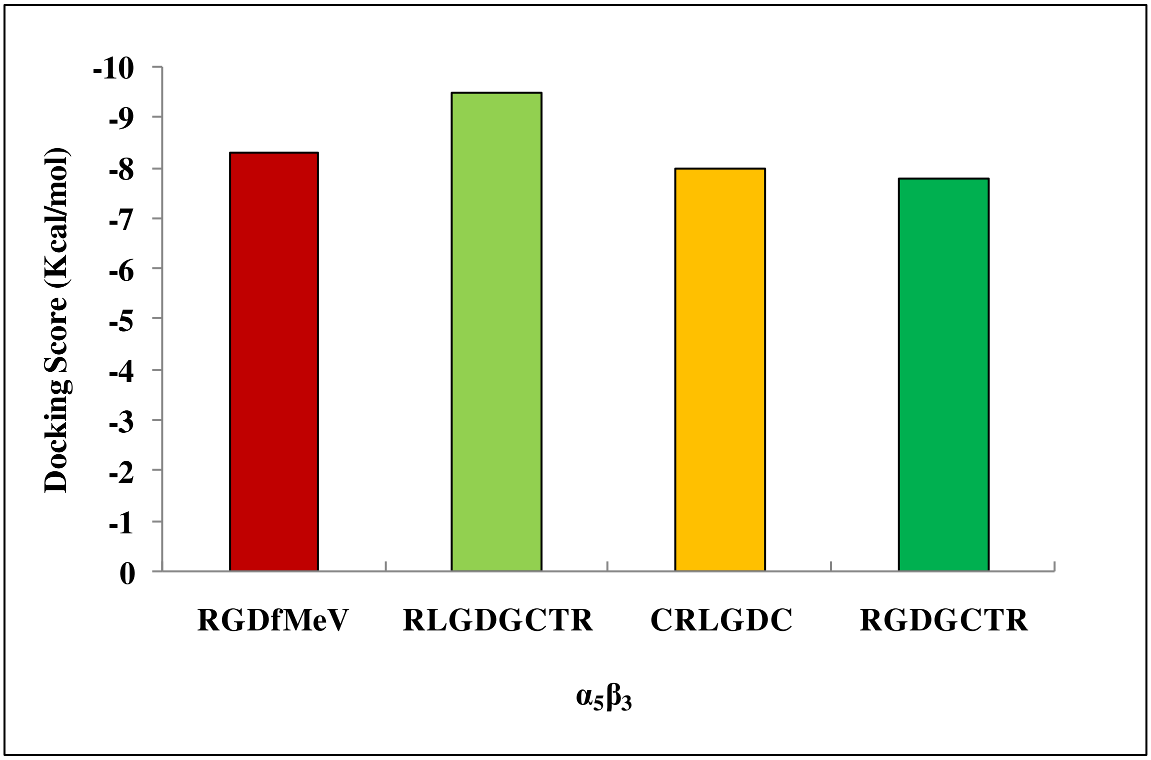

Fig. S4 Relative binding affinities (Kcal/mol) of RGD-like peptides towards integrin $\alpha_5\beta_3$

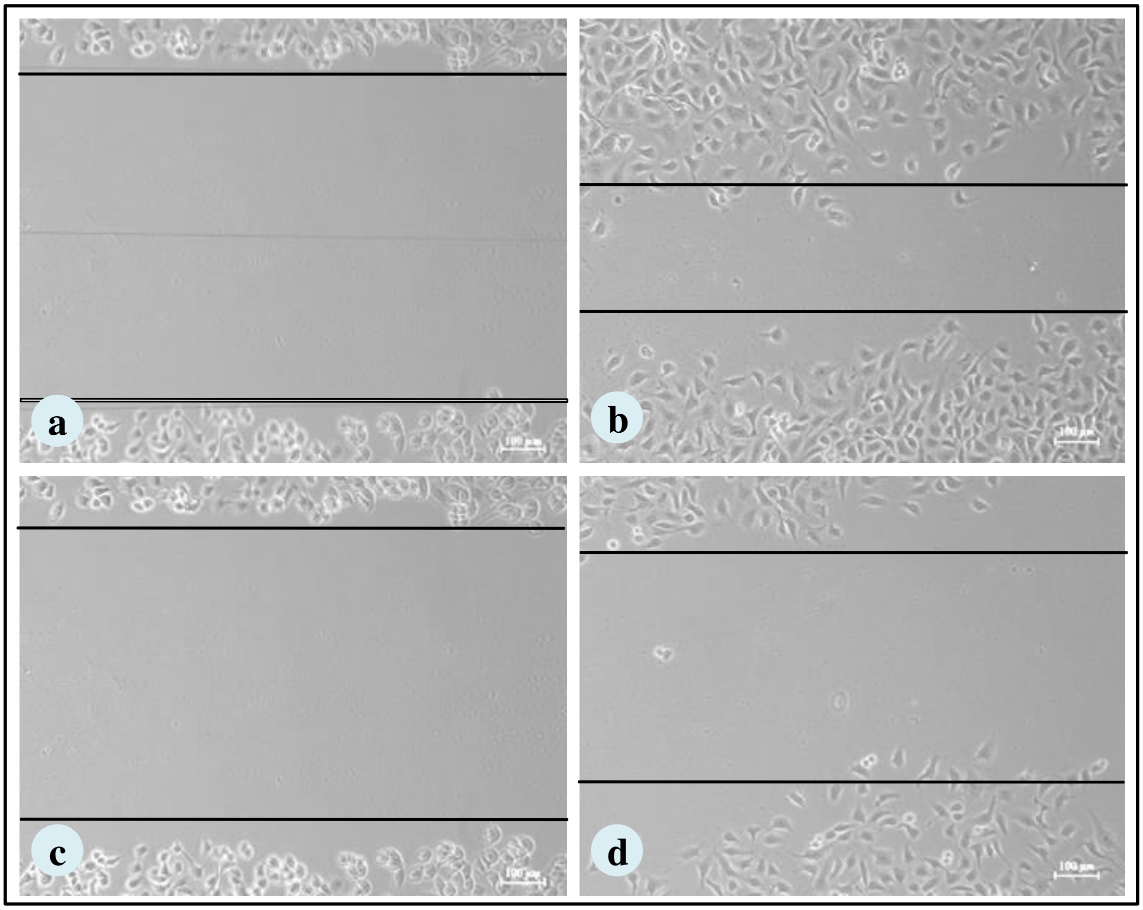

Fig. S5 Scratch assay/Cell migration assay. a) Control MDA-MB-231 cells at 0 h b) Control MDA-MB-231 cells at 12 h c) Cyclosaplin (10 μg/mL) treated MDA-MB-231 cells at 0 h d) Cyclosaplin (10 μg/mL) treated MDA-MB-231 cells at 12 h. Scale bar = 100 μm.

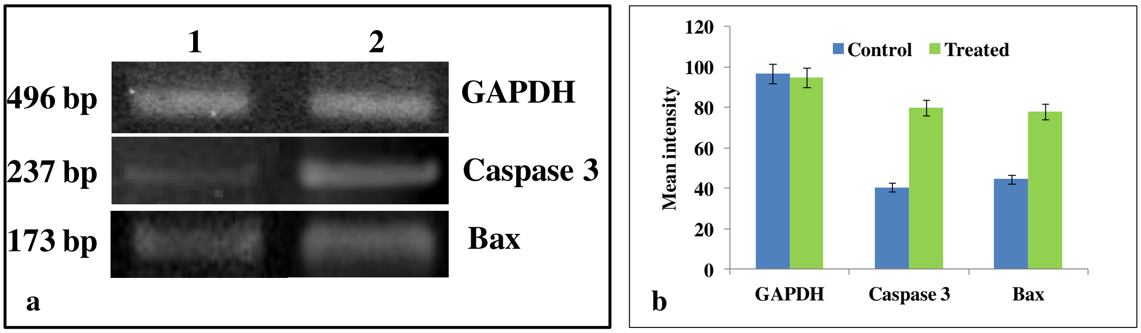

Fig. S6 Reverse transcriptase polymerase chain reaction (RT-PCR) for GAPDH, Caspase 3 and Bax mRNA in cyclosaplin induced MDA-MB-231 cells. a) Lane 1: Control (Untreated), Lane 2: Treated (cyclosaplin, 10 μg/mL) for 24 h. b) Bands were analyzed using image J software and represented as mean intensity (arbitrary units).

Table S1 The binding affinities of cyclosaplin along with its analogues towards integrin $\alpha_5\beta_3$

Peptide	Binding affinities (Kcal/mol)
RGDfMev (Cilengitide)	-8.3
RLGDGCTR (Cyclosaplin)	-9.5
CRLGDC (Analogue I)	-8.0
RGDGCTR (Analogue II)	-7.8
